# Competency- Based Curriculum for Post- graduation in Community Medicine at a Medical College located in western India

**DOI:** 10.15694/mep.2020.000035.2

**Published:** 2021-01-14

**Authors:** Shobha Misra, Vihang Mazumdar

**Affiliations:** 1 P.D.U; 2 P.D.U; 3 Govt. Medical College Baroda; 4 Govt. Medical College Baroda

**Keywords:** Competency-Based Curriculum, Curricular Reforms, Postgraduate, Community Medicine, Needs Assessment Study

## Abstract

This article was migrated. The article was marked as recommended.

**Introduction:** Curriculum or course needs to be monitored and evaluated to ensure that it is working as planned and also to identify areas for improvement. Implementing competency-based training in postgraduate medical education poses many challenges, but ultimately requires a demonstration that the learner is truly competent to progress in training or to the next phase of a professional career.

**Purpose:** The purpose of this document was to design a competency-based curriculum in Community Medicine teaching and training of post graduates so that they could efficiently play their roles as *Community Physician, Public Health Specialist, Health Manager, Faculty, Researcher and Occupational Physician.*

**Methods:** A needs assessment study using cross-sectional design reinforced a strong need of developing a common & structured curriculum in Community Medicine at the department studied and later to be scaled at state level. A Six-Step Approach to Curriculum Development was followed ( Kern *et al.*, 2009) to design and implement the competency based curriculum.

**Results:** Relevant feedback from teachers and students has been incorporated to prepare this document. Almost all students and faculty strongly agreed for the need of introducing formative assessment. They strongly perceived that a formative assessment would be helpful to motivate students and should have weight- age in university exams. It is learnt that there are challenges involved in operationalzing competency-based curriculum viz; requires more time and effort from faculty, at least in the initial phases of development. Other challenges include; finding ways to include internal assessment marks in the university examination.

**Conclusions:** Competency based curriculum is feasible within a conventional medical curriculum. Benefits of such learning include improving knowledge and skills of learner that would lead to improved quality of health care.

## Introduction

A curriculum defines the learning that is expected to take place during a course or program of study in terms of knowledge, skills and attitudes. The written and published curriculum is the official or formal curriculum. Curriculum or course needs to be monitored and evaluated to ensure that it is working as planned and to identify areas of improvement. It is envisaged to increase the pool of competent and skilled specialists and super- specialists so as to cater to the healthcare and educational needs of the rural and urban India and to facilitate every Indian Medical Graduate to be able to pursue post-graduate medical education in India (MCI Vision Document, 2015). Implementing competency-based training in postgraduate (PG) medical education poses many challenges, but ultimately requires a demonstration that the learner is truly competent to progress in training or to the next phase of a professional career.

Community medicine is an academic subject, a branch of medicine that deals with promotion of health and prevention of diseases, involving people’s participation, utilizing professional management skill. A needs assessment study using cross-sectional design reinforced a strong need of developing a common and structured curriculum in Community Medicine at the department and a possibility to be scaled at state level that responds to society’s specific problems ( Misra, 2016). The purpose of this document was to design a competency-based curriculum in Community Medicine teaching and training of post graduates so that they could efficiently play their roles as academician, consultant, researcher, administrator for posts in health services or industries.

## Methods

**Figure 1:**
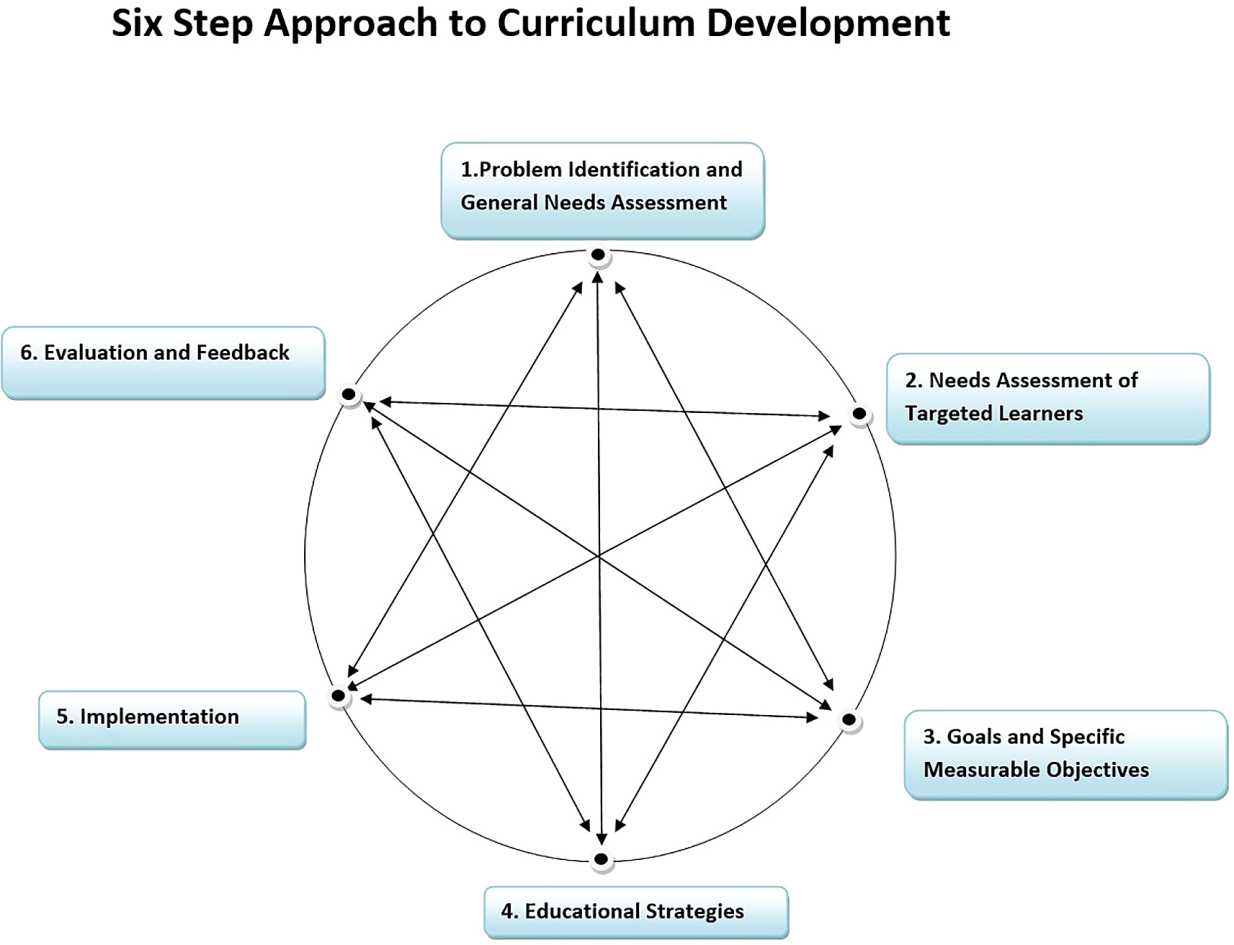
Six-Step Approach to Curriculum Development ( Kern et al., 2009)

The curriculum reforms were designed to be implemented in the department of community medicine of a medical college located in a state of western India. There are six government medical colleges and five self-financed colleges that run the postgraduate (PG) course (MD) in community medicine where more than 50 PGs join this course in a year through a National Eligibility cum Entrance Test (NEET). The course is of three years duration and the purpose of PG education is to create specialists who would provide high quality health care and advance the cause of science through research and training. It is perceived that in the state PG teaching has improved over the years when one to two students were joining the course in various colleges. However, it is also observed by the academicians of community medicine that with the changing scenario of health care in the country reforms are needed to enhance competencies of the learner. A Six-Step Approach to Curriculum Development was followed ( Kern *et al.*, 2009) as shown in Figure 1. And the curriculum is implemented since 2015.

## Results/Analysis

### Six-Step Approach to Curriculum Development ( Kern et al., 2009).

**Step 1:** Problem Identification and General Needs Assessment; A meeting of Professors in Community Medicine ofthe state run medical colleges regarding the required reforms in PG curriculum revealed that; in the present format of postgraduate medical education in Community Medicine the information provided to the students is in disconnected pieces and mostly looks out of context, it encourages mostly a passive approach and pays little emphasis on solving practical problems. A lot of variation in teaching and learning the subject among colleges was also a cause of concern. They suggested that the identified problem could be overcome by designing and implementing a standard curriculum to bring uniformity, while respecting the right of each college to go beyond as per their own needs. Thereafter, a Technical Working Group made specific recommendations relating to curricular reforms which are incorporated in finalizing this document.

**Step 2:** Needs Assessment of the targeted postgraduate students & teachers; A needs assessment study using a Cross-sectional design was conducted through administration of an online questionnaire to identify the need of a common Post Graduate Curriculum ( Misra, 2016). Before formulating the questionnaire, a Focus Group Discussion (FGD) with PG students of the College was conducted. There were ten PGs in the department and all of them were part of one FGD conducted using a semi-structured guideline, developed purposively for the discussion and this was conducted in English. At the beginning of the FGD, the participants were explained regarding the purpose, and confidentiality of the discussion and a verbal consent was taken. They were explained that they had the liberty to refuse to answer any question that they did not like. There were no disturbances and almost all could remain present throughout the discussion. Audiotape was not used as per the denial of the participants. The discussion lasted for one and half hours and the notes were expanded within 24 hrs of the discussion. Transcripts of FGD were prepared by two separate investigators, revised and analysed for content and key concepts as mentioned earlier. The moderator was the first author of this paper who is trained in qualitative research. Based on responses and emerging themes from FGD, the questionnaire was designed and pilot tested on ten PGs. The questionnaire was organized based on the current curriculum, for their opinions about the (1) general demographic information (2) qualities of the current curriculum/needs’ assessment (3) questions requesting classification of syllabus in Must to Know, Nice to Know and Desirable to Know. For questions about the qualities of the curriculum, a five-point agreement scale was used (1-strongly agree, 2-agree, 3-neither agree nor disagree 4-disagree, 5-strongly disagree). In each section of the questionnaire, space was provided for additional comments.

The questionnaire was mailed to 100 PG students and 80 Teachers of Community Medicine from six Government and two private Medical Colleges of the state. Prior approval for the study was obtained from the Institutional Scientific and Ethics Committee. Important findings from needs assessment include;

• Majority of the respondents felt that there were no defined learning outcomes, teaching-learning methods and assessment methods.
• The curriculum lacks clarity, is not Competency-based and lacks linkages with professional development.
• All of them agreed that there is a need of introducing Formative assessment during PG course.

In a teacher’s own words to an open-ended question, “I wondered currently, ‘what a PG student of community medicine does for entire three years? There is a definite need of defining curriculum for three different years”. Another put it like this, “There is no formal formative assessment, except feedback given during presentation”. However, most of them agreed that the available curriculum helped students to utilize basic statistical analysis, material resources were available and students were being supported in independent learning. These findings reinforced a strong need of developing a curriculum for three separate years of course that could be followed by all Medical Colleges of the state. Important and relevant feedback from teachers and students has been incorporated in this document.

**Step 3: Goal and Objectives**; Once the needs of targeted learners were identified, goals and objectives for thecurriculum were written, starting with broad or general goal and then moving towards specific and measurable objectives. Objectives included acquisition of competencies under the three domains of learning viz; Cognitive (knowledge), Affective (attitudinal) & Psychomotor (skill).

**Programme Goal**; *The student shall acquire the understanding and the skill in Community Medicine and develop the quality of leadership so that s/he becomes an expert and function effectively as Community Physician, Public Health Specialist, Health Manager, Faculty and/or Researcher in the field of Community Medicine.*

Some very basicGuidelines for Competency Based PG Training Programme for MD - Community Medicine by Medical Council of India, New Delhi were referred to by the faculty as a team. And first draft of the same was presented at a state level conference.

Entrustable professional activities (EPAs): To produce specialists of Community Medicine who upon successfully qualifying in the M.D examination shall have the following competencies/Skills;

(I) Public Health Specialist; can practice the Community Medicine specialty ethically and in step with the principles of health care.

(II) Epidemiology; has a spirit of scientific enquiry and is oriented to the principles of research methodology and epidemiology.

(III) Health Team Leadership; can function as an effective leader of a health team engaged in health care, research and training.

(IV) Teaching and Training: has the basic skill in training of the medical and paramedical professional.

(V) Research; can demonstrate competence in basic concepts of research methodology.

Entrustable professional activities were then specified in form of departmental objectives for example;

(II) Epidemiology, he/she should be able to;

• effectively use the tools of epidemiology for understanding disease causation and determinants of diseases.
• conduct epidemiological investigation of communicable, non - communicable and other diseases of public health importance and suggest appropriate solution.
• demonstrate competence in basic concepts of research methodology and epidemiology and be able to critically analyze relevant published research literature.

Departmental Objectives and specific learning objectives (SLO) were written in relation to the above competencies for all three domains viz: knowledge, skills and attitude.

**Step 4: Educational Strategies;** Once objectives were specified; curriculum contents were chosen and educationalmethods were selected in alignment with the objectives.

**Course Contents for Theory;** Topic Prioritization Guide was prepared and followed as shown in Table 1 **.** Forguidance to the student and faculty, topics were categorized into three, i) Vital (Must to Know), ii) Essential (nice to Know) & iii) Desirable to know. It gave an idea to the students and faculty regarding, the priority to be given to a topic while teaching and learning.

**Table 1:** Topic Prioritization Guide for Health systems in India and the world - Historical Perspective as an example

MD PSM, course contents for theory; Based on Core Contents	Priority			Teaching Learning Method		
	Must To Know	Nice To Know	Desirable To know	Interactive PG lecture	Discussion	Self-Directed
**1. Health systems in India and the world -Historical Perspective**	√					
1.1. History of Public Health in India	√					
History of Health Services in India	√				√	√
Indigenous Systems of Medicine in India	√				√	√
Bhore Committee’s and other “Committee Reports on Health Services,		√			√	
Health care and Health Professional Education in India.	√					√
National Health Policy	√			√		√
An update of achievements of the country vis-à-vis the Health for all Indicators	√				√	√
1.2. History of Public Health in the World		√				√

**Postings for PG Study in Community Medicine;** Graded and increasing responsibilities were given to the students during first, second and third year of the course as shown in Table 2.

**Table 2:** Postings in Community Medicine at the studied Department

Name of place of posting: External (outside of hospital setting)	Duration in Days/weeks	Level (Semester) *
1. Central Water analysis laboratory/Public Health Laboratory:	Visit for a day under supervision of a faculty	III
2. Infectious Disease Hospital:	Visit for a day under supervision of a faculty	IV
3. Municipal Corporation:	1 week	V
4. Community Health Centre (CHC):(Extension of posting from RHTC)	2 weeks	V, VI
5. CDHO Office	2 week	V
6. Any other	Urban & Rural Health Centers throughout (one year at least during the 3-year period), in rotation with experiential learning.	
**Name of place of posting; Internal (in hospital setting)**	**Duration in Days**	**Level (Terms)**
1. Medicine:	2 weeks	III
2. Pediatrics:	2 weeks	IV
3. Obstetrics and Gynecology	2 weeks	V
OTHERS AS AND WHEN FEASIBLE:		
Foods & Nutrition Dept.	2 days	II
National Institute of Malaria Research (NIMR) NADIAD	A day’s visit with a guide as a teacher	VI
Any other		

* As per local resources and context.

The training was competency based and students were directed to acquire well identified competencies for which a list of competencies was prepared.

**Organization of Teaching and Training:** Teaching and training methods were spread over 6 semesters in threeyears and mostly included; interactive and organized sessions, case studies, practical exercises, guided learning by doing and thesis. All the activities were to be recorded and graded in the Log book. These were summarized at the end of semester/year/course as shown in Table 3.

**Table 3:** List of activities with minimum numbers to be achieved

Sr.	Activities	Minimum Standards/ Numbers held	Achieved (No given or 50% of activities held) Insert T* or A*
1.	Clinical posting (with Microteaching)		15
2.	Field visits		5
3.	Research (Field investigator/assistant)		5
4.	Epidemiological investigation		1
5.	Paper presented (Oral/poster/national/state)		2 in all semesters
6.	Paper published		1 in all semesters
7.	PG Presentation (Attended, presented)		15
8.	Journal Club (Attended, presented)		10
9.	UG Classes (Taken/Attended)		30
10.	Family Study (Carried out/presented)		5
11.	Case study (Clinico-Epidemiolgical /Health System Case study/Hospital /others) Prepared/Presented.		5
12.	UHTC Posting	1 month	1 month
13.	RHTC Posting	1 month	1 month
14.	CHC Posting	1 week	1 week
15.	Health System Office Posting (Meetings attended)	2	2
16.	Health Program Supervision/Monitoring visits	2	2
17.	Epidemiology / Public Health / Biostatistics Exercise	5	5

These were summarized at the end of semester/year/course as below:

Summary of the activities carried out by the PG during __________Semester/_____ year /MD Course

T*= Taken, A*= Attended

Step 5: Implementation; Activities undertaken during;

**First Year:** An Orientation Programme of one week was undertaken so that the students could get acquainted with the department / subject. During first year they got opportunity to review undergraduate (UG) level of learning as all PGs were not at the same level of learning at intake. They were posted at urban and rural health centres to develop competence in performing duties of a basic doctor (MBBS) at the affiliated health centres including referrals. Undertaking basic research methodology workshop, training in Software like EPI Info and SPSS, communicating sympathetically and effectively with patients, relatives and families were important part of training starting right from the first year.

**Second Year:** Application of public health principles of the subject i.e. epidemiology, prevention, environmentalsanitation, nutritional counseling etc. were undertaken. Thesis related proposal writing, field work in form of State Routine Immunization Monitoring (SRIM), epidemic investigations, health department convergence posting, Clinical Posting at other related department like Obstetrics, Pediatrics and Medicine were arranged and conducted. Enhancement of their teaching skill through microteaching sessions was an important aspect of the curriculum. During the second and third year they were required to deliver health care to defined communities in rural and urban settings. They also participated in national health programs and training programs of medical officers and primary health care workers. Data collection for dissertation was completed by second year and the first draft of dissertation was required to be completed before end of 2 ^nd^ year of residency.

**Third Year:** Activities included acquisition of competence in major skills of the subject viz; writing researchproposals and reports, critical appraisal of research papers, participation in national health programs (NHP), health finance/economics, presentation skills, attending meetings conducted by Chief District Health Officer and others. During the third year, emphasis was given to acquisition of skills relating to independent evaluation exercises, organization of surveys and investigation of disease outbreaks and teaching Sessions.

**Teaching Learning Methods;** Formal interactive teaching **,** Problem based learning, Role Play, Panel discussions, Seminar presentations, Microteaching sessions, Observation through visits, Self-Directed Learning through Reflective practice and attending workshops/conferences, Project based learning, Assignments, Demonstrations, Exercises, Videos, Discussion with UGs during Clinical postings and RHTC (UG students and Interns), Teacher and Peer feedback and few others were methods of active learning **.**

**Teaching Schedule;** The departmental teaching schedule included each of the following activity once in everyweek. 1) Journal club/Seminar alternate week. 2) Family / Medico-Social Case Presentation. 3) Epidemiology / Public Health / Biostatistics Exercise. 4) Thesis work discussion / Extra Mural Posting. 5) Field Visit/Microteaching.

A list of Topics for Modular Teaching/Learning was prepared and two of them viz; Integrated Management of Neonatal & Childhood Illness (IMNCI) and Revised National Tuberculosis Control Program (RNTCP) were undertaken as Self-directed learning to start with. List of activities with minimum numbers to be achieved that could be summarized at the end of semester/year/course was also prepared.

### Assessment & Scheme of Examination;

1. Self-Evaluation - Through daily Work Diary and feedback from faculty. 2. Faculty Evaluation - Through scrutiny of Diary (concept maps) and Log Book by Guide and Head of Department. 3. Technique of skills in Pedagogy - Through Microteaching Sessions, lesson plans and supervised taking of clinical posting/tutorial for undergraduates (30 tutorials). 4. Skill Evaluation - Through demonstration, practical and field reports supported by a checklist. 5. Knowledge Evaluation -Through journal clubs, seminars and tests.

**Types of assessment for the proposed curriculum; Formative Assessment:** Formative assessment did not affectresult at the end of the program, but provided feedback to the candidate. A formative assessment three months before the University examination was administered which was as similar as possible to the Summative / university examination. The performance of the Postgraduate student during the training period was monitored throughout the course and was duly recorded in the log books as evidence of the ability and daily work of the student. Annexure /Checklists for Evaluation of various activities were developed.

Marks out of 100 were allotted as below in Table 4.

**Table 4:** Distribution of Marks for Formative Assessment (out of 100)

Sr. No.	Items	Marks	Marks obtained
1	Personal attributes: Behavior and Emotional (Qualitative)	25	
2	Theory, Practical Work and Viva (formative assessment in semesters)	30	
3	Academic activities	25	
4	End of term theory examination (after 2 years and 9 months)	10	
5	End of term practical examination (after 2 years and 9 months)	10	

**Students’ assessment criteria:** A qualitative criteria was well defined for assessment of PG student for Personal Attributes viz: Behaviour and Emotional (for example diligence, regularity, sincerity, teaching skills, interpersonal skills, leadership and motivation and research aptitude) during different academic activities was prepared and was graded in form of a Likert scale of 1-5 (Very poor to Very good) as shown in Table 5.

**Table 5:** Criteria for Assessment of PG Student for Personal attributes: Behavior and Emotional and during different academic activities (Qualitative)

Assessment Criteria (Max 5)	Score *	Remarks
Availability including Punctuality & Regularity		
Interpersonal Skills and Leadership Quality		
Motivation and Initiative		
Diligence including Stability		
Honesty and Integrity		

* Very Good - 5, Good - 4, Average - 3, Poor - 2, Very Poor - 1.

A score of four (institutes can set such standards) in all items be set as an acceptable standard in order to get a final certification for appearing for M.D. University Exam. Marks for personal attributes and academic activity were given annually by all the consultants under whom the resident was posted during the year. Average of the three years to be put as the final marks except end of term exam.

**Summative/University examination:** Was in the form of dissertation/thesis, written papers (Theory), Practical/Clinical and Viva Voce. As per university requirement a PG student is allowed to appear in the university examination only after the acceptance of the thesis by two of the three examiners appointed by the university. A Sample of practical examination is depicted in Table 6. It is planned to redistribute topics in the 4 theory papers and this shall be put up in the forthcoming Board of studies meeting of the University. Attempt was made to frame Modified Essay type (MET) and Patient Vignette type questions as this helps to cover wide contents of the curriculum and is likely to improve the validity of the testing method.

**Table 6:** A Sample of MD (Community Medicine) University Practical Examination at the studied Department

Particular	Number	Marks	Time Duration
Long family case from the community	1	150	45 minutes
Short Case	2 (One of them includes Inspection of Public Health Practices of public health importance and other from a communicable disease)	150 (75 each)	30 minutes each
Public Health Spots	5-10	50	5 minutes each
Epidemiological + Statistical problem-solving exercises	3 Epidemiological and 2 statistical	50 (25 each)	45-60 minutes
Viva with or without Pedagogy Exercise		200	
TOTAL		600	

**Step 6: Feedback and Evaluation:** Reflections of students in the form of feedback were obtained to improve uponthe course, which is now being implemented regularly for other enrolled PG students. The first batch of PGs trained through this structured curriculum have now been absorbed as Lecturers in medical college (n=2), Medical Officers (with administrative roles in urban corporation and rural area, n=2) and as Public Health experts (UNICEF and Taluka Health Office of a city, n=2).

One of the students who underwent the course reflected as “A Needs Assessment in form of Focus Group Discussion for the PGs regarding their perceptions about the curriculum and expectations from the teaching staff was carried out; based on which, topics to be taken as part of teaching were decided. Cadre-wise faculty was assigned to undertake various topics for PG teaching”. Emphasis was given on Healthcare Administration and Qualitative Research.

Another one mentioned that “The PGs were given teaching sessions for the MBBS students (Clinical Postings) after ***‘Microteaching*** *’*sessions to improve their teaching skills. Peer based learning and case-based learning session wereheld for Health Management topics. Case based learning approach was also used for Community based E-learning. The innovative aspect was structuring rural training and planning of skits/ plays at Rural Health Training Centre (RHTC) which helped us to improve interactive and communication skills”.

**Figure 2:**
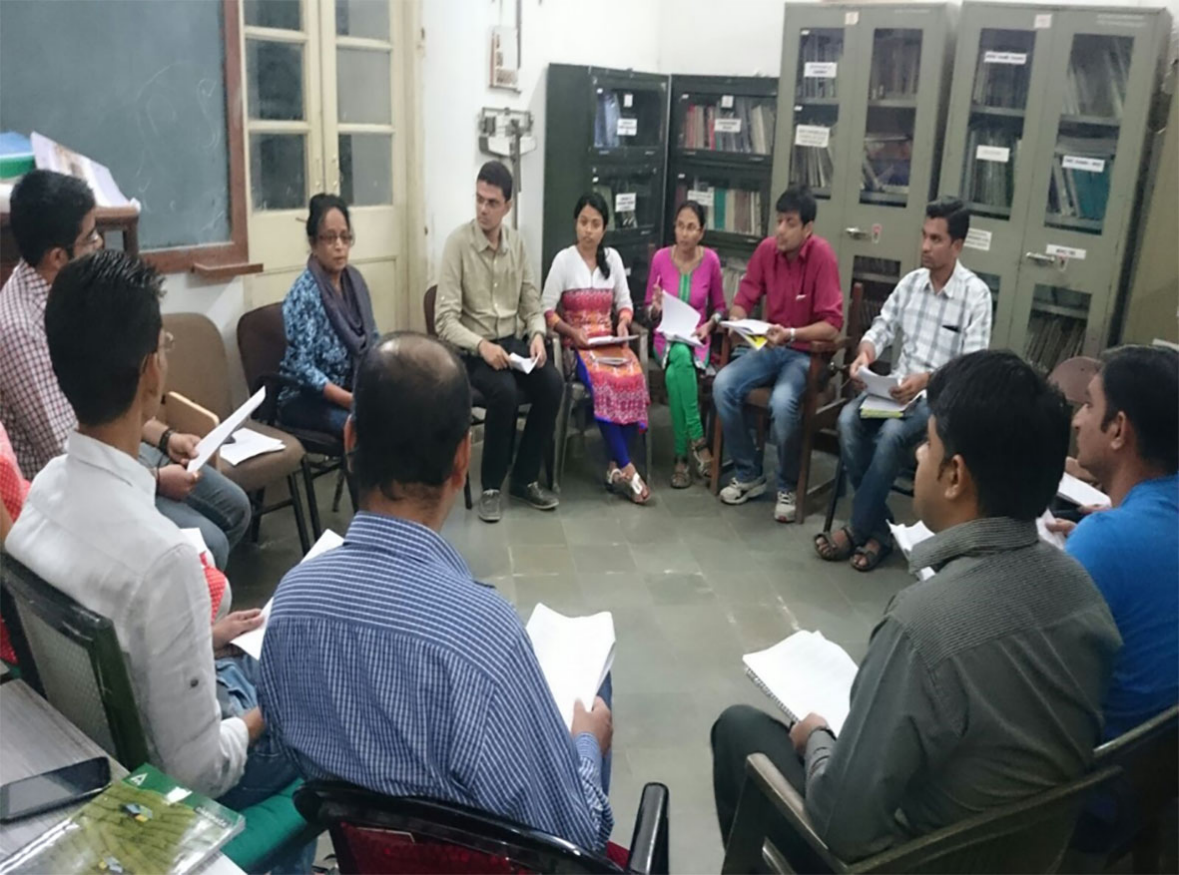
Informal discussion with postgraduate students regarding the Curriculum

**Program evaluation:** Informal observations / discussions with students & faculty to provide ongoing feedback wereundertaken. The purpose was to bring improvement in the curriculum and the learners. Also, to gain support and resources for the curriculum, in research situations to answer questions about the effectiveness of the curriculum and relative merits of the different educational approaches, Figure 2.

Majority of the teachers and students mentioned that the curriculum was in alignment with the learning outcomes, teaching- learning methods and assessments. Most of them felt that the teaching and learning methods were relevant to the content and learning outcomes.

The curriculum did help students to apply knowledge to clinical/field situations and initiated critical thinking and problem-solving skills in them was mentioned by most of them. Majority of them agreed that resources were available to ensure effective teaching and learning for the curriculum content. The faculty made efforts to observe and certify core competencies.

All of the students and faculty strongly agreed that there is a need of introducing formative assessment during PG course. And that formative assessment would be helpful in motivating students and the same should have weight-age in final exams. Evaluation through a semi-structured questionnaire is planned with faculty and students of the forthcoming batch.

## Discussion

**Lessons learnt:** It is learnt that the Competency-based curriculum highlights benefits, is feasible and a valuablevehicle for acquiring competencies in all three domains viz; knowledge, skills and attitude. Based on faculty feedback, this provided a common platform and enhanced coordination between different faculties. We also learned that there are challenges involved in operationalization viz; it requires more time and effort from faculty, at least in the initial phases of curriculum development. Other challenges include; finding ways to include the internal assessment marks in the university examination and ensuring better student participation throughout the program. Inclusion of in-depth qualitative feedback from faculty and students would yield better insight into scaling of the program. In summary, we learned that this type of competency-based curriculum is feasible within a conventional medical curriculum. The benefits of such learning include improving knowledge and skills of learners, and hence are likely to improve quality of health care provided to the patients.

## Conclusion

**What next?** The long-term purpose of this document is to standardize Community Medicine teaching and training of post graduate throughout the state by organizing a dissemination seminar for faculty, professional and regulatory bodies and members of the university’s board of studies committee. Enlisting support and feedback from different colleges of the state would result in achieving uniformity in postgraduate teaching of the subject. We are sure this Technical Document will go a long way in revamping the Community Medicine subject not only in the state but throughout all colleges of India.

## Take Home Messages

• It is learnt that the Competency-based curriculum highlights benefits and is a valuable vehicle for acquiring competencies in all three domains viz; knowledge, skills and attitude.
• There are challenges involved in operationalization viz; it requires more time and effort from faculty, at least in the initial phases of curriculum development. Other challenges include; finding ways to include internal assessment marks in the university examination.
• We learned that this type of competency based curriculum is feasible within a conventional medical curriculum.

## Notes On Contributors

**Dr. Shobha Misra**, Professor & Head, Dept. of Community Medicine, PDU, Govt. Medical College Rajkot, Gujarat, India. M.D. (PSM), DHA, NDDY, PGDMRCHP, Grad Certificate in Clinical Investigation (USF), Certificate HRMH, FAIMER FELLOW (Philadelphia), FIAPSM.

**Dr. Vihang Mazumdar**, Professor & Head, Dept. of Community Medicine, Govt. Medical College Baroda, Gujarat, India. MD Community Medicine.
