# Supplementary material for: Competency- Based Curriculum for Post- graduation in Community Medicine at a Medical College located in western India
Source: MedEdPublish (2016). 2021 Jan 14;9:35. Originally published 2020 Feb 20. [Version 2] doi: 10.15694/mep.2020.000035.2 (PMC10697439; doi:10.15694/mep.2020.000035.2)
Supplement: Peer review history

# Reviewer report: Reviewer response for version 2

**DOI:** 10.21956/mep.20261.r31442

**Published:** 2021-05-15

**Authors:** Madalena Folque Patricio

**Affiliations:** 1 School of Medicine, University of Lisbon

This review has been migrated. The reviewer awarded 5 stars out of 5 Congratulations to the authors for this excellent paper. When I say ‘excellent’ I am thinking not only on the value of what was implemented but also on the way the paper was written, giving all details of what was done which allows readers to replicate this initiative if they wish.Let me highlight some very positive aspects namely the fact that:• Goals and objectives were based on the results of needs assessment • Competency-based curriculum objectives included the three domains of cognitive, affective, and psychomotor learning (described in Bloom’s 1956 taxonomy) • A curriculum mapping approach was implemented starting by defining goals and objectives (based on needs assessment) and then moving towards specific and measurable objectives • Formal and informal evaluation of the programme has been implemented• Microteaching, feedback and learners’ self-assessment were introduced in the programme• The Six-Step Approach to Curriculum Development (Kern et al., 2009) was adopted.My only suggestion would be to add a 7th step to Kern et al. approach,, namely, to introduce the reformulation and/or implementation of changes (based on 6th step of evaluation and feedback), before starting a new cycle, according to the approach of ‘learning, reflection and change’ proposed by Donald Schön already in 1983. To conclude I would say that it was with great satisfaction that I also saw the ambitious and challenging sustainability plan stated by the authors, that is, ‘to standardize post graduate teaching/training Community Medicine across India, obtaining support and feedback from diﬀerent state colleges to achieve uniformity. (Here I dare to propose the achieving harmonization instead of uniformity).Therefore this paper is extremely important to show that this type of competency-based curriculum is feasible within a conventional medical curriculum. Let me conclude by wishing a great success to the authors namely to achieve their ambitious and challenging sustainability plan across India

Reviewer Expertise:

NA

No decision status is available

# Reviewer report: Reviewer response for version 2

**DOI:** 10.21956/mep.20261.r31443

**Published:** 2021-04-10

**Authors:** Ken Masters

**Affiliations:** 1 Sultan Qaboos University

This review has been migrated. The reviewer awarded 5 stars out of 5 This is a revised version of an already-strong paper. I am pleased to see that the authors have taken the Version 1 reviewers’ comments into account, and have addressed most of their concerns.The Discussion is rather shorter than I would have liked, but, as this was not an issue pointed out by the reviewers, it is fine.Well done on a useful piece of writing.

Reviewer Expertise:

NA

No decision status is available

# Reviewer report: Reviewer response for version 1

**DOI:** 10.21956/mep.19000.r27254

**Published:** 2020-02-27

**Authors:** P Ravi Shankar

**Affiliations:** 1 American International Medical University

This review has been migrated. The reviewer awarded 3 stars out of 5 Thank you for the opportunity to review an interesting manuscript. The authors have introduced many innovative teaching learning methods in community medicine and are to be commended. Their approach to curriculum development has also been described well. Based on the title I was expecting more information on the competencies required from a postgraduate in community medicine. What are the overall competencies of the program? What competencies are expected at the end of the first, second and third year? The authors have provided an overview but specific details about these competencies and how they will be assessed and what is an acceptable level will be of interest. How will these competencies be assessed? The authors may also have considered entrustable professional activities (EPAs) which can fit in very well into the scheme of training. How were the competencies required from a postgraduate developed by the authors? They have provided some information but I may be interested in more details. Also many authors insist that formative assessment is mainly for providing feedback and guidance to students and teachers, and the grades be not included in the final assessment. I would be interested in the details of this interesting project. The article will be of general interest to educators and of special interest to educators in community medicine.

Reviewer Expertise:

NA

No decision status is available

# Reviewer report: Reviewer response for version 1

**DOI:** 10.21956/mep.19000.r27252

**Published:** 2020-02-26

**Authors:** Felix Silwimba

**Affiliations:** 1 University of Lusaka

This review has been migrated. The reviewer awarded 5 stars out of 5 thanks for sharing. this paper is well explained and educative .

Reviewer Expertise:

NA

No decision status is available

# Reviewer report: Reviewer response for version 1

**DOI:** 10.21956/mep.19000.r27253

**Published:** 2020-02-24

**Authors:** BALAJI ARUMUGAM

**Affiliations:** 1 TAGORE MEDICAL COLLEGE AND HOSPITAL

This review has been migrated. The reviewer awarded 4 stars out of 5 Dear Dr. Shobha Misra, Congrats to the effort taken to prepare a competency based curriculum for post graduates to be implemented at the state level in future. I have few questions in this step: A needs assessment study using a Cross-sectional design was conducted through administration of an online questionnaire to identify the need of a common Post Graduate Curriculum. Before formulating the questionnaire, a Focus Group Discussion (FGD) with PG students of the College was conducted using a guideline. Based on their responses and peer review, the questionnaire was designed. The same was mailed to 100 PG students and 80 Teachers of Community Medicine from six Government and two private Medical Colleges of the state.1. How many FGDs were conducted?2. How many PGs were involved in each FGD?3. Details of the FGDs and faculty (as moderator) involved was not mentioned?4. Standardization process of the needs assessment questionnaire? 5. Findings of the cross sectional design of needs assessment can be shared (more)?

Reviewer Expertise:

NA

No decision status is available
